# Evaluating the prognostic significance of the modified prognostic nutritional index—C-reactive protein-to-albumin-to-lymphocyte index in acute decompensated heart failure: special attention to the impact of diabetes

**DOI:** 10.3389/fnut.2025.1636685

**Published:** 2025-11-28

**Authors:** Na Zhang, Shuhua Zhang, Lin Xie, Hengcheng Lu, Qun Wang, Zhiyu Xiong, Zhiting Wu, Jinyan Zhang, Yafei Jian, Wanfen Huang, Yinghao Kuang, Xinfang Huang, Wei Wang, Yang Zou, Hongyi Yang

**Affiliations:** 1 Department of Endocrinology, Jiangxi Provincial People’s Hospital, The First Affiliated Hospital of Nanchang Medical College, Nanchang, Jiangxi, China; 2 Jiangxi Cardiovascular Research Institute, Jiangxi Provincial People’s Hospital, The First Affiliated Hospital of Nanchang Medical College, Nanchang, Jiangxi, China; 3 Discipline Construction Office, Jiangxi Provincial People’s Hospital, The First Affiliated Hospital of Nanchang Medical College, Nanchang, Jiangxi, China

**Keywords:** C-reactive protein-to-albumin-to-lymphocyte, prognostic nutritional index, acute decompensated heart failure, mortality, glycemic status, diabetes

## Abstract

**Objective:**

Malnutrition is one of the most common complications in acute decompensated heart failure (ADHF). This study investigated the predictive value of a modified prognostic nutritional index (PNI)—the C-reactive protein-to-albumin-to-lymphocyte (CALLY) index—for short-term mortality in ADHF patients, while accounting for the potential interactive effects of participants’ glycemic status.

**Method:**

The data were derived from the Jiangxi-ADHF II study cohort, which included 1,225 ADHF patients. The Boruta algorithm was employed to identify key prognostic features associated with mortality in ADHF and rank their predictive importance. Subsequently, multivariate Cox regression analysis and receiver operating characteristic curve analysis were conducted to evaluate and compare the prognostic significance of the PNI and CALLY index in predicting short-term mortality in ADHF patients. Exploratory subgroup analyses, including diabetes subgroups, were performed to assess the generalizability of these findings across populations.

**Results:**

During the 30-day observation period, 109 (8.9%) participants experienced mortality. Using the Boruta algorithm, the CALLY index was identified as a key factor associated with ADHF-related mortality. In mortality risk assessment, the CALLY index demonstrated a stronger inverse association with mortality risk in ADHF patients compared to PNI. Quartile-based analysis revealed significantly higher mortality risks associated with low CALLY index relative to low PNI (HR: Q1 4.21 vs. 3.32). For mortality outcome prediction, the CALLY index (AUC = 0.80) was significantly superior to the PNI. Exploratory subgroup analyses further revealed that glycemic metabolic status may act as a significant interaction term in the association between the CALLY index and short-term prognosis in ADHF: compared to non-diabetic ADHF patients, those with comorbid diabetes exhibited a stronger inverse association between the CALLY index and 30-day mortality risk. This finding implies that diabetes significantly amplifies the mortality risk associated with low CALLY index.

**Conclusion:**

In conclusion, the CALLY index, modified based on the PNI, serves as a valuable prognostic tool for short-term outcomes in ADHF patients, with special attention required regarding the potential inhibitory effect of diabetes status on the CALLY index. The promotion of early risk stratification awareness and implementation of CALLY index screening in ADHF patients should be encouraged, particularly in those with comorbid diabetes.

## Graphical abstract

**Figure:**
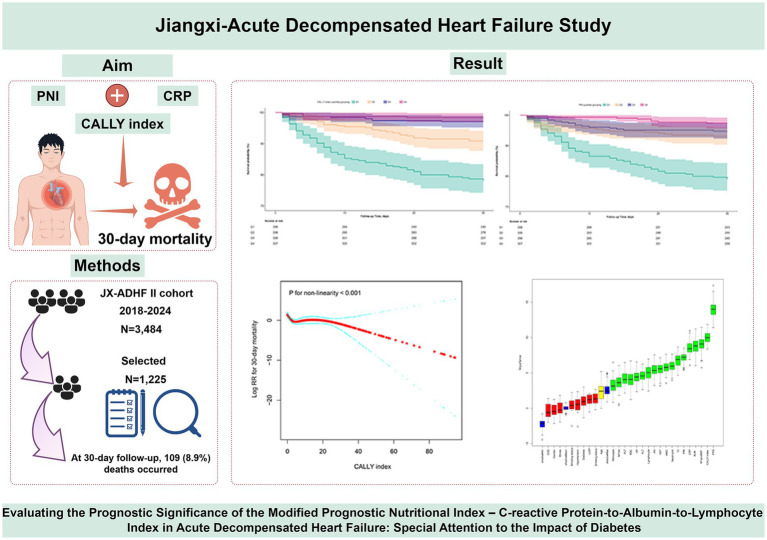

## Background

Acute decompensated heart failure (ADHF) is one of the leading causes of hospitalization worldwide, characterized by complex pathophysiological mechanisms and poor short-term prognosis (1, 2). Malnutrition represents one of the most common comorbidities in ADHF, significantly influencing disease trajectory (3). Studies indicate that approximately 75–90% of ADHF patients suffer from malnutrition (4–6), and its severity is positively correlated with adverse prognosis (3, 7, 8). Current HF management guidelines explicitly integrate nutritional assessment and individualized nutritional interventions into components of standardized care (9). Consequently, various nutritional assessment tools have been developed to facilitate early risk stratification for disease progression and prognostic evaluation (10, 11).

The prognostic nutritional index (PNI) is a simple nutritional assessment tool calculated by combining peripheral blood lymphocyte count and serum albumin (Alb) levels (12). Numerous clinical studies have confirmed its prognostic utility across multiple disease contexts, including ADHF (13–18). However, it is noteworthy that the PNI fails to adequately account for systemic acute inflammation (19), which constitutes a pivotal factor in the pathophysiology of ADHF (2, 20–22). The C-reactive protein-to-albumin-to-lymphocyte (CALLY) index represents a recently developed modification of the PNI (23). By incorporating C-reactive protein (CRP) as a supplementary explanation for systemic inflammation, this index expands the original PNI framework. Relevant evidence highlights its significant prognostic value across diverse clinical contexts, including various tumor diseases (24–26), acute and chronic metabolic disorders (acute stroke and diabetes) (27, 28), immune-mediated diseases (29), and critical illnesses (30, 31). Furthermore, recent studies suggest the CALLY index may serve as a predictive index for long-term outcomes in coronary heart disease (CHD) and heart failure with preserved ejection fraction (HFpEF) (32–34). These findings position the CALLY index—a composite nutritional-inflammatory biomarker—as a promising new tool for cardiovascular prognostic evaluation. According to the study by He et al. (34), the CALLY index was identified as an independent predictor of adverse prognosis in elderly patients with HFpEF, with an accuracy exceeding 75% for predicting long-term survival outcomes. However, it should be noted that current evidence remains unclear regarding whether the CALLY index demonstrates comparable prognostic utility for short-term outcomes in HF patients, and whether it exhibits superior predictive performance compared to the original PNI in this context. In addition, as one of the most common types of HF, ADHF is associated with poor short-term prognosis, which remains one of the greatest challenges for clinicians (1, 2). Therefore, it is necessary to employ simple and effective indicators for risk stratification in this population at an early stage. Based on the above background, the present study aims to evaluate and compare the prognostic value of both the PNI and CALLY index for short-term outcomes in ADHF patients using data from the Jiangxi-ADHF II cohort, to provide evidence-based references for the early risk stratification of ADHF.

## Methods

### Study population and design

The Jiangxi-ADHF II cohort represents a physician-initiated retrospective study. Its primary objectives are to maximize the utility of clinical data from hospital records of ADHF patients, explore novel methodologies for early risk stratification in this population, and generate valuable research evidence for improving adverse clinical outcomes. The Jiangxi-ADHF II cohort consecutively enrolled 3,484 hospitalized patients diagnosed with ADHF at Jiangxi Provincial People’s Hospital between January 2018 and January 2024. Diagnostic criteria followed the then-current European Society of Cardiology guidelines for acute and chronic heart failure management, with the latest version available at admission serving as reference. Regarding study design, the study protocol received formal ethical approval from the Ethics Committee of Jiangxi Provincial People’s Hospital (No. 2024-01). Regarding data utilization, written informed consent was obtained from all participants or their legally authorized representatives. The study complied with the ethical principles outlined in the Declaration of Helsinki and adhered to the Strengthening the Reporting of Observational Studies in Epidemiology guidelines for reporting observational research findings.

**Figure 1:**
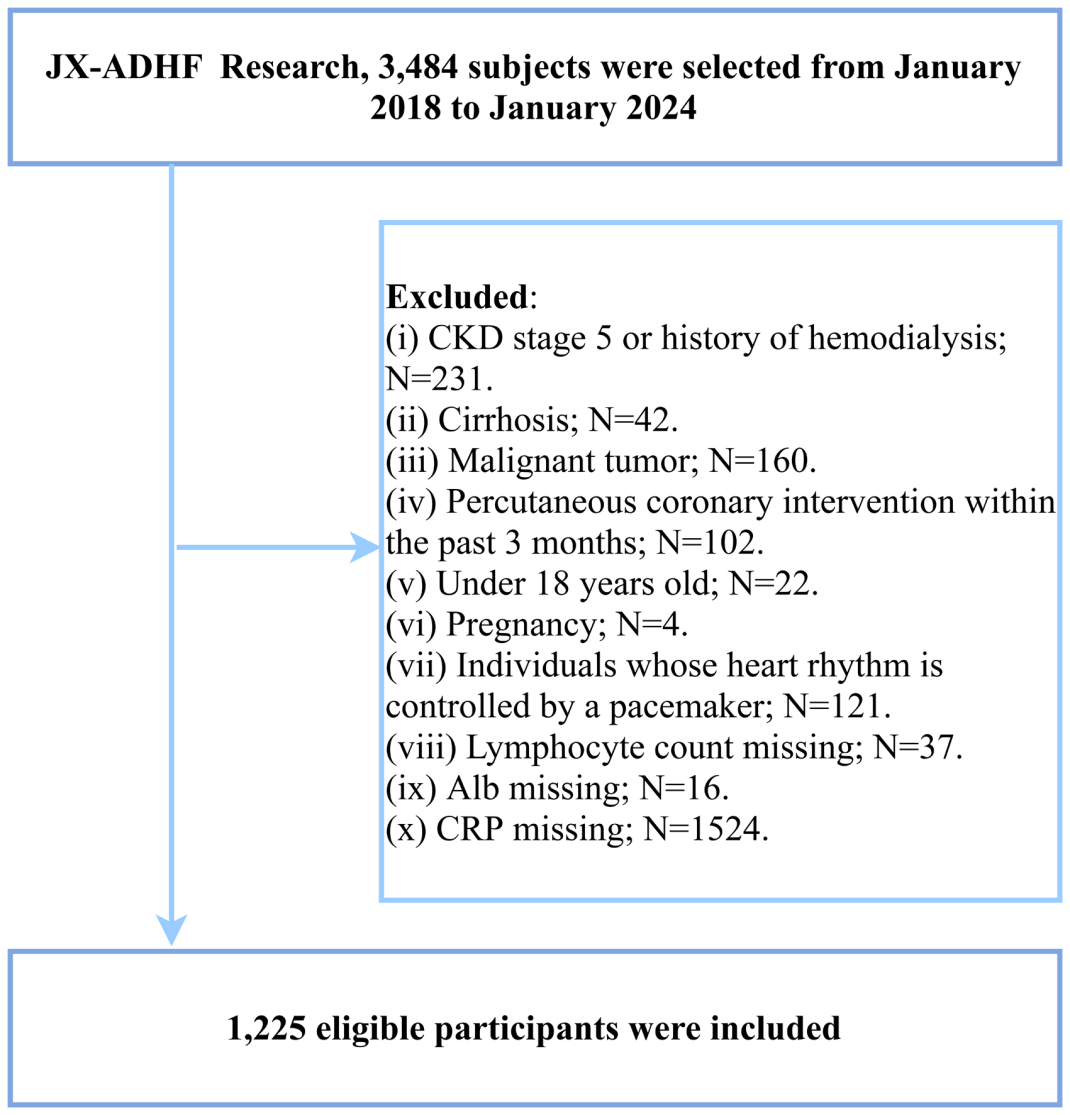
Flow chart for inclusion and exclusion of study participants.

In the current study, we aimed to investigate the association between the CALLY index and short-term outcomes in ADHF patients. The participant screening workflow and implementation process are summarized in Figure 1 and outlined as follows: (1) We excluded subjects with significant fluid and sodium retention secondary to non-cardiac conditions, including patients with liver cirrhosis, uremia, and those with chronic kidney disease undergoing hemodialysis treatment (*n* = 273). (2) Patients with malignancies were excluded, as their limited life expectancy could significantly impact the study outcomes (*n* = 160). (3) Given the potential confounding effect of reperfusion therapy on short-term prognosis, individuals who had undergone percutaneous coronary intervention within 3 months prior to enrollment were excluded (*n* = 102). (4) Patients with implanted cardiac pacemakers were excluded due to potential autonomic nervous system dysregulation (*n* = 121). (5) Pregnant women and minors (aged <18 years) were also excluded (*n* = 26). (6) Participants with missing CALLY index data were excluded (*n* = 1,577).

### Assessment of covariates

We evaluated baseline data of participants within 24 h of admission, encompassing the New York Heart Association (NYHA) functional classification assessed at admission, sociodemographic characteristics (age and gender), lifestyle habits (drinking and smoking status), personal medical history (hypertension, diabetes, stroke, and CHD), and echocardiographic parameters—specifically left ventricular ejection fraction (LVEF).

Venous blood samples were collected by nursing professionals after admission and transported to the Medical Laboratory Center of Jiangxi Provincial People’s Hospital, where they were analyzed using automated analyzers by trained laboratory technicians. The panel of routine blood tests and biochemical markers included: white blood cell count, neutrophil count, monocyte count, lymphocyte count, red blood cell count (RBC), platelet count (PLT), Alb, creatinine (Cr), blood urea nitrogen (BUN), alanine aminotransferase, aspartate aminotransferase (AST), fasting plasma glucose (FPG), CRP, and N-terminal pro-B-type natriuretic peptide (NT-proBNP).

### Assessment of PNI and CALLY index


$$\mathrm{PNI}=\mathrm{Alb}\;(g/L)+5\times \text{lymphocyte count}\;(10^{9}/L)$$

$$\text{CALLY index}=[\begin{array}{l} \mathrm{Alb}\;(g/L)\times \text{lymphocyte count}\;(\text{cells}/\mathrm{\mu L})\\ /(\mathrm{CRP}\;(\mathrm{mg}/\mathrm{dL})\times 10^{4}) \end{array}]$$

### Determination of study outcomes

This study primarily assessed the 30-day all-cause mortality rate among ADHF patients following hospitalization. Using the admission date as the reference time point (Day 0), participants’ survival status and the occurrence date of the endpoint event (death) were systematically recorded throughout the 30-day follow-up period.

### Handling of missing data

As CRP testing is not routinely performed in ADHF patients, this observational study consequently excluded many participants due to missing CRP values. To enhance methodological transparency, we further compared the baseline characteristics between participants with and without CRP measurements in this study. The comparison results (Supplementary Table 1; most *p*-values >0.05) indicated that the majority of baseline characteristics showed similar distributions between the missing and non-missing CRP groups. These findings suggest that the missing CRP data occurred randomly and were independent of both observed and unobserved factors.

In the current study, partial missing values were observed for the covariates LVEF, Cr, BUN, and FPG, with a maximum missing rate of 3.75% (detailed in Supplementary Table 2). Given the relatively low proportion of missing data, the study retained the original dataset for analysis to preserve data authenticity and minimize potential bias.

### Statistical analysis

R (version 4.2.1) and Empower^®^ (version 4.2) statistical software were utilized for data analysis in this study, and a two-tailed significance level of 5% was employed. Baseline characteristics of the study population are presented as frequency (percentage), mean ± standard deviation, or median and interquartile range.

**Figure 2:**
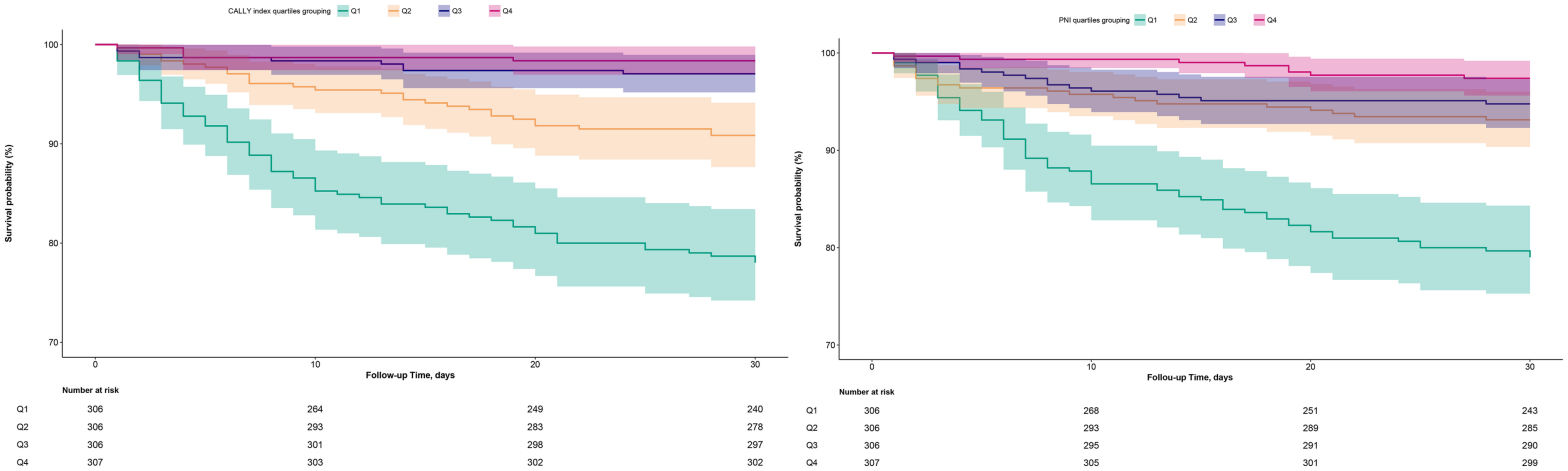
30-day survival curves of ADHF patients stratified by PNI and CALLY index quartiles. ADHF, acute decompensated heart failure; PNI, prognostic nutritional index; CALLY, C-reactive protein-to-albumin-to-lymphocyte.

This study employed the Boruta algorithm for feature selection, an all-relevant feature selection method based on random forests. Unlike traditional minimal-optimal feature selection methods (e.g., LASSO regression) that aim to identify the smallest feature subset for optimal prediction, the Boruta algorithm offers significant advantages in nonlinear modeling, handling mixed-type variables, resilience to multicollinearity, and operational stability (35–37). These capabilities allow it to identify all relevant features. By comparing the importance of original features against a set of randomly generated “shadow” features, Boruta can robustly determine whether each original feature has a true association with the outcome and rank the key features. This makes it particularly suitable for highly complex, multi-variable interaction scenarios such as healthcare (35–37). Three multivariate-adjusted Cox regression models were constructed to evaluate the associations of the CALLY index and PNI with mortality in ADHF patients. Covariates included in the models were gender, age, hypertension, diabetes, stroke, CHD, NYHA classification, drinking status, smoking status, LVEF, neutrophil count, monocyte count, RBC, PLT, AST, Cr, BUN, UA, FPG, and NT-proBNP. To ensure comparability of the associations between PNI and CALLY index with mortality, we further evaluated the relationship between quartile groups of both indices and study outcomes, calculating corresponding hazard ratios (HRs) and 95% confidence intervals. Notably, all included covariates passed multicollinearity testing (Supplementary Tables 3, 4). Additionally, visual assessment of survival curves associated with PNI and the CALLY index (Figure 2) confirmed the applicability of the proportional hazards assumption.

Following confirmation of the roles of the PNI and CALLY index in 30-day mortality risk assessment, we further constructed receiver operating characteristic curves to systematically assess and compare the predictive performance of these indices and their constituent components for 30-day mortality events in ADHF patients. Corresponding area under the curve (AUC) values, specificity, sensitivity, and optimal thresholds were calculated. In addition, we further compared the predictive performance of the CALLY index, the PNI, and the established ADHERE (Acute Decompensated Heart Failure National Registry) risk score for short-term mortality (38). The AUC and net reclassification improvement were calculated to evaluate their incremental predictive performance.

We employed a 4-knot restricted cubic spline to model the dose–response relationship between the CALLY index and 30-day mortality risk in ADHF patients. A likelihood ratio test was used to examine potential nonlinear effects. Upon detecting a nonlinear association, we employed recursive algorithms to identify inflection points where risk significantly changed. Subsequently, piecewise Cox regression models were constructed to quantify the strength of associations between the CALLY index and short-term mortality risk in ADHF patients on either side of these inflection points.

Finally, we conducted stratified analyses to evaluate the association between the CALLY index and 30-day mortality risk in ADHF patients. Stratification factors included age (grouped by median value), gender, LVEF (categorized using a 50% cutoff), and comorbidities (hypertension, diabetes, stroke, and CHD). Given that multiple subgroup comparisons were conducted in this study, we applied the Bonferroni correction to control for the inflation of type I errors caused by multiple comparisons. A total of 14 independent tests were involved in this analysis; therefore, the statistical significance threshold for subgroup results was set at *p* < 0.0036. Likelihood ratio tests were applied to compare differences across strata and assess the presence of interaction effects.

### Sensitivity analysis

To test the robustness of our findings, we further evaluated the association between the CALLY index and 30-day mortality risk in ADHF patients under various priori assumptions:

(1) Considering the potential nonlinear age effect, a squared term for age was incorporated into the final analytical model (39).
(2) Multimorbidity significantly contributes to frailty and adverse outcomes (40, 41). To mitigate this potential confounding effect, we excluded patients with three or more concurrent chronic conditions (hypertension, diabetes, stroke, and CHD) in the current analysis.
(3) To control for potential reverse causation, we re-evaluated the association between the CALLY index and 30-day mortality risk after excluding patients who died within the first 3 days of follow-up.
(4) Multiple imputation was applied to address missing data; the association between the CALLY index and 30-day mortality risk in ADHF patients was reassessed using the imputed complete dataset.

## Results

### Baseline characteristics

Table 1 displays the baseline characteristics of the study population stratified by CALLY index quartiles. The median age of participants was 71 years, with a male predominance (58.04% vs. 41.96%). The cohort predominantly comprised individuals from Jiangxi Province, China. Compared to participants with higher CALLY index scores, those with lower scores were generally older, had a higher prevalence of chronic comorbidities (CHD, diabetes, stroke, and hypertension), were more likely to be male and smokers, and exhibited higher levels of CRP, white blood cell count, neutrophil count, monocyte count, AST, BUN, Cr, UA, FPG, NT-proBNP, alongside lower levels of RBC and lymphocyte count (Table 1).

**Table 1:** Summary of baseline characteristics of the study population according to CALLY index quartile groups.

Variable	CALLY index quartiles				*p*-value
	Q1 (0.01–0.85)	Q2 (0.86–3.81)	Q3 (3.82–11.10)	Q4 (11.12–242.18)	
No. of subjects	306	306	306	307	
Age (years)	74.00 (64.00–81.75)	72.00 (63.00–81.00)	70.00 (59.00–77.00)	66.00 (54.00–75.00)	<0.01
Gender (*n*, %)					<0.01
Male	204 (66.67%)	181 (59.15%)	177 (57.84%)	149 (48.53%)	
Female	102 (33.33%)	125 (40.85%)	129 (42.16%)	158 (51.47%)	
Hypertension (*n*, %)					0.06
No	188 (61.44%)	158 (51.63%)	168 (54.90%)	183 (59.61%)	
Yes	118 (38.56%)	148 (48.37%)	138 (45.10%)	124 (40.39%)	
Diabetes (*n*, %)					0.07
No	223 (72.88%)	216 (70.59%)	222 (72.55%)	244 (79.48%)	
Yes	83 (27.12%)	90 (29.41%)	84 (27.45%)	63 (20.52%)	
Stroke (*n*, %)					0.88
No	254 (83.01%)	247 (80.72%)	252 (82.35%)	254 (82.74%)	
Yes	52 (16.99%)	59 (19.28%)	54 (17.65%)	53 (17.26%)	
CHD (*n*, %)					0.09
No	207 (67.65%)	210 (68.63%)	217 (70.92%)	234 (76.22%)	
Yes	99 (32.35%)	96 (31.37%)	89 (29.08%)	73 (23.78%)	
NYHA classification (*n*, %)					<0.01
III	176 (57.52%)	186 (60.78%)	202 (66.01%)	238 (77.52%)	
IV	130 (42.48%)	120 (39.22%)	104 (33.99%)	69 (22.48%)	
Drinking status (*n*, %)					0.96
No	279 (91.18%)	278 (90.85%)	277 (90.52%)	276 (89.90%)	
Yes	27 (8.82%)	28 (9.15%)	29 (9.48%)	31 (10.10%)	
Smoking status (*n*, %)					0.16
No	245 (80.07%)	251 (82.03%)	263 (85.95%)	262 (85.34%)	
Yes	61 (19.93%)	55 (17.97%)	43 (14.05%)	45 (14.66%)	
LVEF (%)	51.00 (40.00–57.00)	48.00 (37.75–56.00)	47.00 (37.00–57.00)	51.00 (40.00–58.00)	0.04
CRP (mg/L)	79.10 (47.60–120.75)	14.98 (10.30–23.08)	5.87 (4.40–8.39)	2.04 (1.34–3.04)	<0.01
WBC (×10^9^/L)	7.70 (5.55–11.18)	6.54 (5.10–8.47)	6.32 (4.84–8.00)	5.80 (4.82–7.05)	<0.01
Neutrophil count (×10^9^/L)	6.00 (4.10–9.88)	4.92 (3.60–6.49)	4.31 (3.20–6.00)	3.65 (2.88–4.77)	<0.01
Lymphocyte count (×10^9^/L)	0.63 (0.40–0.95)	0.91 (0.62–1.30)	1.10 (0.80–1.47)	1.40 (1.02–1.80)	<0001
Monocyte count (×10^9^/L)	0.50 (0.36–0.70)	0.52 (0.40–0.70)	0.50 (0.38–0.62)	0.47 (0.35–0.60)	<0.01
RBC (×10^12^/L)	3.73 (0.84)	3.95 (0.78)	4.09 (0.73)	4.24 (0.75)	<0.01
PLT (×10^9^/L)	167.00 (130.25–229.75)	165.50 (127.25–222.00)	174.50 (129.25–216.75)	165.00 (134.00–202.50)	0.50
Alb (g/L)	31.41 (5.35)	33.68 (4.69)	35.86 (4.49)	37.57 (4.12)	<0.01
ALT (U/L)	21.00 (13.00–41.00)	23.00 (14.00–42.00)	22.00 (13.00–39.00)	23.00 (16.00–37.00)	0.62
AST (U/L)	29.50 (20.00–49.00)	27.50 (20.00–46.75)	25.00 (19.00–37.00)	26.00 (20.00–35.50)	<0.01
Cr (μmol/L)	100.00 (75.00–157.00)	95.00 (76.00–130.00)	87.00 (67.00–117.00)	76.50 (62.25–97.00)	<0.01
BUN (mmol/L)	9.38 (6.73–14.07)	8.10 (6.11–11.08)	7.04 (5.31–9.90)	6.42 (5.07–8.12)	<0.01
UA (umol/L)	424.00 (320.00–540.00)	447.00 (348.00–567.00)	431.50 (336.25–537.00)	397.00 (310.00–474.00)	<0.01
FPG (mmol/L)	5.80 (4.80–6.80)	5.40 (4.70–6.40)	5.40 (4.80–6.20)	5.20 (4.60–6.00)	<0.01
NT-proBNP (pmol/L)	4390.50 (1956.75–7754.00)	4100.00 (1863.25–7911.25)	3272.50 (1715.50–6105.50)	2703.00 (1283.00–4444.50)	<0.01

CHD, coronary heart disease; NYHA, New York Heart Association; LVEF: left ventricular ejection fraction; Cr, creatinine; WBC, white blood cell count; RBC, red blood cell count; PLT, platelet count; ALT, alanine aminotransferase; AST, aspartate aminotransferase; NT-proBNP, N-terminal pro-brain natriuretic peptide; UA, uric acid; CRP, C-reactive protein; Alb, albumin; FPG, fasting plasma glucose; CALLY index, C-reactive protein-to-albumin-to-lymphocyte index.

### Follow-up outcomes

During a median observation period of 30 days, 109 (8.9%) participants experienced mortality events. Mortality rates stratified by CALLY index quartiles were 21.90, 9.15, 2.94, and 1.63% while those for PNI quartiles were 20.92, 6.86, 5.23, and 2.61%. Kaplan–Meier analysis further visualized the 30-day survival curves for CALLY index and PNI quartile groups, revealing that patients in the lower CALLY index groups (Q1 and Q2) exhibited relatively worse survival outcomes compared to the lower PNI groups (Q1 and Q2) (Figure 2).

### Feature importance ranking via Boruta algorithm for 30-day mortality in ADHF patients

**Figure 3:**
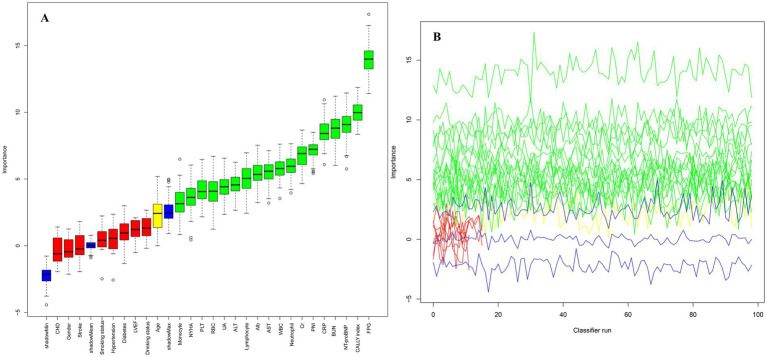
Feature selection for 30-day mortality in ADHF patients using the Boruta algorithm. **(A)** The process of feature selection. **(B)** The value evolution of the *Z*-score in the screening process. The horizontal axis shows the name of each variable and the number of times the classifier is run in **A,B**, respectively. The vertical axis represents the *Z*-value of each variable. The green boxes and lines represent confirmed variables, the yellow ones represent tentative attributes, and the red ones represent rejected variables in the model calculation.

This study employed the Boruta algorithm for feature selection, utilizing shadow features as reference benchmarks to systematically compare the *Z*-scores of actual features against those of shadow features. Variables with significantly higher *Z*-scores than shadow features were labeled green (critical features), while those without significant differences were labeled red (non-critical features). Variables falling in the yellow zone represented tentative factors. Using the Boruta algorithm, we identified 18 variables most significantly associated with 30-day mortality risk in ADHF patients (Figure 3, green zone). The analysis revealed that the CALLY index emerged as one of the most influential factors affecting 30-day mortality in ADHF patients, exhibiting higher predictive importance than the PNI and ranking second only to FPG.

### Observational associations of CALLY index, PNI, and 30-day mortality in ADHF patients

Table 2 summarizes the associations between the CALLY index, PNI, and 30-day mortality in ADHF patients. The study demonstrated inverse associations between both the CALLY index and PNI with 30-day mortality risk in ADHF patients across unadjusted and adjusted models: Specifically, each unit increase in the CALLY index was associated with an 8% reduction in 30-day mortality risk (HR 0.92, 0.86–0.97); similarly, each unit increase in PNI corresponded to a 6% risk reduction (HR 0.94, 0.91–0.97). To better quantify the impact of the CALLY index and PNI on 30-day mortality risk in ADHF patients, we calculated the mortality risks stratified by quartiles of these parameters. The results demonstrated that compared to the highest CALLY index quartile (Q4), patients in the lowest quartile (Q1) exhibited a 321% increased risk of 30-day mortality (HR 4.21, 1.59–11.13). Similarly, compared to the highest PNI quartile (Q4), those in the lowest PNI quartile (Q1) had a 232% increased risk (HR 3.32, 1.42–7.77). In summary, lower CALLY index and lower PNI were independent risk factors for short-term mortality risk in ADHF patients, with lower CALLY index conferring a posing mortality risk than lower PNI.

**Table 2:** Multivariable Cox regression analysis of the association between PNI, CALLY index, and 30-day mortality in patients with ADHF.

Independent variable	30-day mortality	Hazard ratios (95% confidence interval)			
		Unadjusted Model	Model I	Model II	Model III
CALLY index		0.83 (0.78, 0.89)	0.84 (0.79, 0.90)	0.85 (0.79, 0.91)	0.92 (0.86, 0.97)
CALLY index quartiles					
Q4	5 (1.63%)	1.0	1.0	1.0	1.0
Q3	9 (2.94%)	1.82 (0.61, 5.42)	1.64 (0.55, 4.90)	1.17 (0.38, 3.59)	0.92 (0.29, 2.87)
Q2	28 (9.15%)	5.78 (2.23, 14.98)	4.80 (1.84, 12.50)	3.98 (1.52, 10.38)	2.40 (0.90, 6.40)
Q1	67 (21.90%)	14.99 (6.04, 37.19)	12.83 (5.12, 32.11)	9.46 (3.74, 23.97)	4.21 (1.59, 11.13)
PNI		0.88 (0.85, 0.90)	0.88 (0.86, 0.91)	0.89 (0.86, 0.92)	0.94 (0.91, 0.97)
PNI quartiles					
Q4	8 (2.61%)	1.0	1.0	1.0	1.0
Q3	16 (5.23%)	2.05 (0.88, 4.78)	1.81 (0.77, 4.24)	1.74 (0.71, 4.29)	1.83 (0.73, 4.61)
Q2	21 (6.86%)	2.72 (1.20, 6.13)	2.13 (0.93, 4.87)	1.98 (0.83, 4.73)	1.46 (0.59, 3.61)
Q1	64 (20.92%)	8.86 (4.25, 18.48)	6.91 (3.27, 14.59)	6.15 (2.76, 13.69)	3.32 (1.42, 7.77)

PNI, prognostic nutritional index; CALLY, C-reactive protein-to-albumin-to-lymphocyte; ADHF, acute decompensated heart failure.

Model I adjusted for gender, age, hypertension, diabetes, stroke, and CHD.

Model II adjusted for model I + NYHA classification, drinking status, smoking status, and LVEF.

Model III adjusted for Model II + neutrophil count, monocyte count, RBC, PLT, AST, Cr, BUN, UA, FPG, NT-proBNP.

### Predictive performance of PNI, CALLY index, and their components for 30-day mortality in ADHF patients

**Figure 4:**
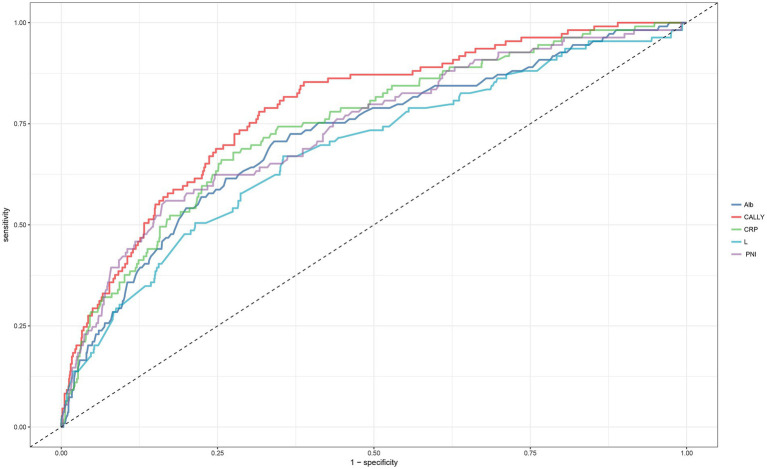
Receiver operating characteristic curve analysis was performed to assess the predictive performance of PNI, the CALLY index, and its constituent parameters for 30-day mortality in patients with ADHF. ADHF, acute decompensated heart failure; PNI, prognostic nutritional index; CALLY, C-reactive protein-to-albumin-to-lymphocyte.

We evaluated the predictive accuracy of PNI, the CALLY index, and their constituent components for 30-day mortality in ADHF patients by calculating the AUC, sensitivity, specificity, and optimal thresholds. As shown in Table 3, the CALLY index demonstrated the highest predictive accuracy for short-term mortality events compared to PNI and individual components of the CALLY index (Figure 4; AUC = 0.80), with an optimal threshold determined to be 2.79.

**Table 3:** Area under the receiver operating characteristic curve of the PNI, CALLY index and its components on 30-day mortality in patients with ADHF.

Variable	AUC	95% CI low	95% CI up	Best threshold	Specificity	Sensitivity
Alb^a^	0.72	0.66	0.77	33.45	0.66	0.70
Lymphocyte count^a^	0.68	0.63	0.74	0.84	0.64	0.67
CRP^a^	0.75	0.70	0.79	23.65	0.74	0.66
PNI^a^	0.74	0.68	0.79	34.83	0.83	0.56
CALLY index	0.80	0.74	0.83	2.79	0.61	0.85

AUC, area under the curve; other abbreviations as in Table 1.

a DeLong *p* < 0.05, compared with CALLY index.

### Comparison of the predictive value of the CALLY index model, PNI model, and ADHERE model for mortality

As shown in Supplementary Tables 5, 6, we compared the predictive performance of the CALLY index model against the PNI and ADHERE models. The analysis revealed that the CALLY index model achieved an AUC of 0.80, significantly outperforming the PNI (AUC = 0.74) and ADHERE (AUC = 0.61) models (DeLong test, *p* < 0.05). Continuous net reclassification analysis demonstrated that, compared with the PNI model and ADHERE model, the CALLY index model achieved a significant net improvement, with a net reclassification improvement greater than 0.2 (*p* < 0.05).

### Dose–response relationship between CALLY index and 30-day mortality in ADHF patients

**Figure 5:**
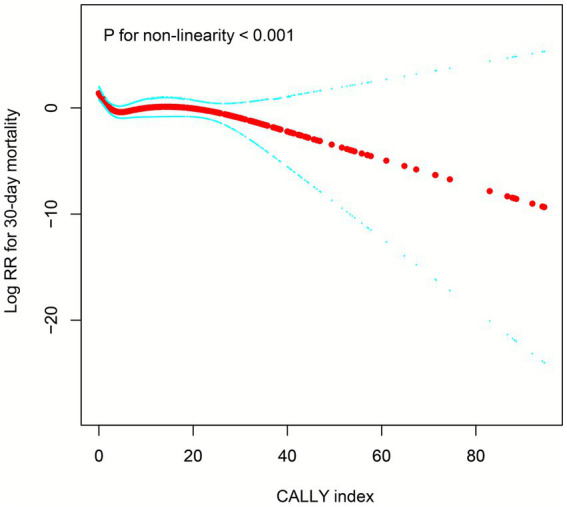
Fitting the dose–response relationship between CALLY index and 30-day mortality in ADHF patients with four knots restricted cubic spline. CALLY, C-reactive protein-to-albumin-to-lymphocyte; ADHF, acute decompensated heart failure. Adjusted for gender, age, hypertension, diabetes, stroke, CHD, NYHA classification, drinking status, smoking status, LVEF, neutrophil count, monocyte count, RBC, PLT, AST, Cr, BUN, UA, FPG, NT-proBNP.

We further constructed a restricted cubic spline curve to visualize the association between the CALLY index and 30-day mortality risk in ADHF patients (Figure 5). The results revealed a strong correlation between low CALLY index levels and elevated 30-day mortality risk. Notably, nonlinearity testing demonstrated a significant nonlinear association between the CALLY index and 30-day mortality in ADHF patients (*p* for nonlinearity <0.001). This nonlinear pattern was further characterized by a saturating effect (L-shaped pattern), where 30-day mortality risk plateaued after the CALLY index surpassed a certain threshold. Through recursive algorithm, we identified an inflection point at 3.14 for the association between the CALLY index and 30-day mortality risk, with segmented Cox regression analyses (Table 4) revealing distinct risk patterns across this threshold: pre-inflection (CALLY index <3.14), each unit increase was associated with a 39% reduction in mortality risk (HR 0.61, 0.49–0.75), whereas post-inflection (CALLY index >3.14), the risk reduction became non-significant at 1% (HR 0.99, 0.95–1.03).

**Table 4:** The result of the two-piecewise Cox regression model.

Independent variable	Hazard ratios (95% confidence interval) *p*-value
The inflection point of the CALLY index	3.14
<3.14	0.61 (0.49, 0.75) < 0.01
>3.14	0.99 (0.95, 1.03) 0.51
*p* for likelihood test	<0.01

CALLY, C-reactive protein-to-albumin-to-lymphocyte.

### Stratified analysis of CALLY index-mortality association across subgroups

We conducted exploratory subgroup analyses to evaluate the association between the CALLY index and 30-day mortality in ADHF patients. Subgroups were stratified by age, gender, LVEF, and comorbidities (hypertension, diabetes, stroke, and CHD). The findings demonstrated that the association between the CALLY index and 30-day mortality in ADHF patients remained robust in the majority of subgroups (Table 5). However, after further Bonferroni correction (for 14 comparisons), a significant association between the CALLY index and 30-day risk was observed only in ADHF patients with diabetes (*p* < 0.0001). In addition, further interaction tests revealed a significant difference in the CALLY index-associated 30-day mortality risk among the diabetic subgroup (*p*-interaction <0.01): Compared to ADHF patients without diabetes, those with diabetes exhibited a significantly stronger inverse association between the CALLY index and short-term mortality outcomes (HR: diabetes 0.73 vs. nondiabetic 0.95). This suggests that diabetes significantly amplifies the mortality risk associated with low CALLY index scores in ADHF patients.

**Table 5:** Stratified analysis showed the relationship between CALLY index and 30-day mortality in patients with ADHF in different age, gender, NYHA classification, LVEF, and whether combined with hypertension/diabetes/cerebral stroke/CHD.

Subgroup	Adjusted HR (95% CI)	Bonferroni-corrected *p*-value	*p* for interaction
Age (years)			0.52
19–70	0.89 (0.79, 1.00)	0.0439	
71–99	0.93 (0.86, 0.99)	0.0349	
Gender			0.99
Male	0.92 (0.85, 0.99)	0.0274	
Female	0.92 (0.83, 1.02)	0.0975	
LVEF			0.84
< 50%	0.91 (0.83, 0.99)	0.0344	
≥ 50%	0.92 (0.85, 1.00)	0.0426	
Hypertension			0.55
Yes	0.93 (0.85, 1.00)	0.0640	
No	0.89 (0.82, 0.98)	0.0156	
Diabetes			<0.01
Yes	0.73 (0.59, 0.92)	<0.0001	
No	0.95 (0.90, 1.01)	0.0881	
Stroke			0.55
Yes	0.95 (0.85, 1.05)	0.2944	
No	0.91 (0.84, 0.98)	0.0102	
CHD			0.64
Yes	0.96 (0.91, 1.03)	0.2430	
No	0.86 (0.77, 0.95)	0.0042	

Abbreviations are as in Table 1.

Models adjusted for the same covariates as in model III (Table 2), except for the stratification variable.

### Robustness assessment of the association between CALLY index and 30-day mortality in ADHF patients

We further conducted age-adjusted nonlinear analyses, special population analyses, temporal sensitivity analyses, and data integrity assessments to evaluate the robustness of the association. The robustness of the association between the CALLY index and 30-day mortality in ADHF patients was confirmed, remaining largely unchanged even after incorporating an age-squared term (Table 6: sensitivity-1). After controlling for potential reverse causation, the association pattern between the CALLY index and ADHF patients remained unchanged (Table 6: sensitivity-2). Following additional adjustment for frailty as a potential confounder, the primary findings remained robust with no substantive alterations (Table 6: sensitivity-3). Finally, replication of the primary analysis in a multiple-imputed complete dataset confirmed the results’ robustness (Table 6: sensitivity-4).

**Table 6:** Sensitivity analysis.

Independent variable	Hazard ratios (95% confidence interval)			
	Sensitivity-1	Sensitivity-2	Sensitivity-3	Sensitivity-4
CALLY index	0.92 (0.86, 0.97)	0.89 (0.82, 0.96)	0.91 (0.85, 0.97)	0.91 (0.86, 0.97)
CALLY index quartiles				
Q4	Ref	Ref	Ref	Ref
Q3	0.89 (0.29, 2.80)	0.77 (0.20, 2.96)	0.53 (0.15, 1.94)	1.03 (0.34, 3.12)
Q2	2.45 (0.92, 6.52)	2.74 (0.91, 8.25)	1.98 (0.70, 5.58)	2.37 (0.89, 6.32)
Q1	4.17 (1.58, 11.02)	4.82 (1.62, 14.35)	3.80 (1.37, 10.53)	4.67 (1.78, 12.23)

Sensitivity-1: The final model included a quadratic term for age.

Sensitivity-2: Excluded participants who died within 3 days after admission.

Sensitivity-3: Patients defined as frail were excluded.

Sensitivity-4: Multiple imputation was used to handle missing data, and the association analysis was repeated.

Adjusted for gender, age, hypertension, diabetes, stroke, CHD, NYHA classification, drinking status, smoking status, LVEF, neutrophil count, monocyte count, RBC, PLT, AST, Cr, BUN, UA, FPG, NT-proBNP.

Hypertension, diabetes, stroke, and CHD were not adjusted in sensitivity-3.

## Discussion

This cohort study investigating the prognostic utility of the CALLY index in ADHF patients demonstrates that the CALLY index is a superior tool for assessing short-term prognosis compared with the PNI. Furthermore, our findings highlight the L-shaped dose–response relationship between the CALLY index and short-term mortality risk in ADHF patients and the significant modifying effect of diabetes.

The CALLY index, a modified version of the PNI, is a novel nutritional-inflammatory biomarker developed by Iida and colleagues in recent years (23). It incorporates peripheral blood lymphocyte count, Alb, and CRP. Compared to the PNI, the CALLY index further integrates CRP, an acute-phase inflammatory protein that serves as an early inflammatory biomarker in ADHF patients (42–44). Prior studies have validated the CALLY index’s significance in risk assessment across multiple chronic conditions, including chronic obstructive pulmonary disease (45), asthma (46), stroke (47), erectile dysfunction (48), angina (49), metabolic syndrome (50), sarcopenia (51), and cardiorenal syndrome (52). Furthermore, the CALLY index demonstrates broad applicability in prognostic assessment across various neoplastic diseases (24–26), acute and chronic metabolic conditions (including acute stroke and diabetes) (27, 28), immune-mediated disorders (29), critical illnesses (30, 31), and cardiovascular diseases (32–34, 53, 54). Collectively, these findings underscore the CALLY index’s potential as a versatile nutritional-inflammatory biomarker with considerable clinical utility and translational value. In summary, a low CALLY index exerts significant detrimental effects on overall physiological health. In the context of HF prognosis assessment, recent evidence demonstrates that the CALLY index serves as an independent predictor of long-term outcomes in elderly patients with HFpEF. He’s et al. (34) longitudinal study involving 320 participants revealed a significant inverse association between the CALLY index and long-term prognosis in this population (HR = 0.81) after adjusting for BNP, BUN, antiplatelet agents, angiotensin II receptor blockers, and statins. In this study, we specifically analyzed the association between the CALLY index and short-term outcomes in ADHF patients. After adjusting for gender, age, hypertension, diabetes, stroke, CHD, NYHA classification, drinking status, smoking status, LVEF, neutrophil count, monocyte count, RBC, PLT, AST, Cr, BUN, UA, FPG, and NT-proBNP, our results demonstrated a significant inverse association between the CALLY index and short-term prognosis in ADHF patients, with superior risk stratification capability compared to the PNI. Furthermore, subgroup analyses revealed no significant differences in this association between patients with HFpEF and those with reduced ejection fraction. Compared to the study by He et al. (34), the current investigation involved distinct HF populations and a substantially larger sample size, enabling more extensive subgroup exploratory analyses. Overall, this research significantly expands the evidence base for the CALLY index’s utility in short-term cardiovascular prognostic assessment and conclusively demonstrates its superiority over the PNI as a prognostic marker for short-term risk stratification in ADHF.

In recent years, the clinical utility of the CALLY index in predicting mortality has garnered significant attention. The CALLY index demonstrates predictive accuracy ranging from 63 to 83% for 1- to 5-year survival in gastrointestinal malignancies (55–60), with particularly high accuracy in predicting overall survival following radical resection of intrahepatic cholangiocarcinoma (55). For patients with chronic obstructive pulmonary disease, the CALLY index demonstrates 59% accuracy in predicting 5-year mortality and 66% accuracy for 10-year mortality (61). Additionally, several observational studies from China and Turkey have reported the CALLY index’s long-term prognostic performance in cardiovascular patients. A Turkish study demonstrated that the CALLY index predicts 3-year mortality in acute coronary syndrome patients with approximately 67% accuracy (53). Similar findings were replicated in Chinese populations, Ji et al. (33) reported even higher predictive accuracy (82%) for 3-year mortality in patients with ST-segment elevation myocardial infarction. Notably, a recent report by He et al. (34) involving elderly patients with HFpEF demonstrated that the CALLY index predicts 1-, 3-, and 5-year mortality with high accuracy (77, 75, and 78% respectively) and remarkable temporal stability. In the current study, our analysis of ADHF patients revealed that the CALLY index exhibits approximately 80% predictive accuracy for short-term mortality, modestly outperforming the medium- to long-term performance observed in He’s et al. (34) cohort. Furthermore, we conducted an additional analysis of the CALLY index’s predictive performance for 30-day mortality in elderly patients with HFpEF, demonstrating an improved predictive accuracy of 82% (Supplementary Table 7). In summary, these findings underscore the CALLY index’s robust prognostic accuracy for mortality prediction in Chinese cardiovascular disease patients and warrant further investigation.

The exact pathophysiological mechanisms underlying the association between the CALLY index and short-term mortality prognosis in ADHF patients remain largely unknown. However, based on the methodology of the CALLY index, we hypothesize that nutritional and inflammatory factors may independently or synergistically contribute to adverse outcomes in ADHF patients. ADHF is a clinical syndrome resulting from various etiologies, characterized by either new-onset heart failure or acute deterioration of chronic heart failure. Its primary features include acute dyspnea and fluid and sodium retention, often accompanied by gastrointestinal edema and congestion (1, 2). When gastrointestinal congestion occurs, nutrient absorption and utilization become significantly impaired (62), potentially leading to intestinal lymphocytic loss and subsequent compromise of cardiac function (63, 64). Furthermore, during the acute phase of HF, significant activation of inflammatory pathways occurs, accompanied by the systemic release of numerous inflammatory mediators, including CRP, tumor necrosis factor-α, interleukin-6, interleukin-1, galectin-3, and soluble suppression of tumorigenicity 2 (20–22). These mediators not only mediate lymphocyte apoptosis (65, 66) but also stimulate the secretion of catabolic hormones such as glucagon, cortisol, and catecholamines, thereby exacerbating malnutrition (67–69). It is noteworthy that activation of inflammatory pathways is often accompanied by increased nutritional demands; if nutritional intake is inadequate, this may compromise immune defense function, thereby elevating the risk of adverse outcomes. Similarly, persistent inflammation can exacerbate the development of malnutrition, creating a bidirectional vicious cycle (70, 71). Moreover, during HF exacerbation, neurohormonal systems are similarly activated, further promoting lymphocyte apoptosis and contributing to immune dysregulation (72). In patients with ADHF, inflammation and malnutrition may mutually influence each other, forming a vicious cycle that ultimately elevates the risk of short-term mortality.

It is worth mentioning that in the current study, we also observed population-specific dependencies within the diabetic subgroup. Regarding this particular finding, and considering the clinical implications of the CALLY index, we hypothesize that this phenomenon may be associated with diabetes exacerbating inflammatory burden and worsening nutritional status in HF. First, it is essential to clarify that the pathophysiological link between diabetes and HF involves multiple mechanisms, including metabolic disorders rooted in insulin resistance (e.g., glucotoxicity, lipotoxicity), vascular endothelial dysfunction, microcirculatory disorders, and microvascular dysfunction (73). (1) Inflammatory perspective: Diabetes, as a complex constellation of metabolic disorders, exacerbates cardiac inflammatory responses through modulation of multiple pathways (74–78). (2) Nutritional perspective: Metabolic disturbances induced by diabetes may serve as a critical determinant of systemic nutrient depletion through several mechanisms: (i) Persistent hyperglycemia promotes substantial urinary glucose excretion, resulting in energy wastage (79). (ii) Insulin resistance may contribute to reduced protein synthesis and enhanced protein degradation (80, 81). Additionally, elevated levels of proinflammatory cytokines in diabetes may directly mediate muscle catabolism (81). (iii) Gastrointestinal complications may induce malabsorption: Studies demonstrate that chronic hyperglycemia and insulin resistance can compromise digestive system function, thereby impairing the digestion and absorption of nutrients (82, 83). (3) Diabetes-related inflammation and malnutrition interact to create a detrimental positive feedback loop (70, 71, 84–86).

In clinical practice, the CALLY index is particularly suitable for implementation in healthcare facilities at all levels due to its testing simplicity and cost-effectiveness. Although a relatively high proportion of HF patients in clinical practice cannot be assessed with the CALLY index due to the lack of CRP testing, the CALLY index has demonstrated good predictive performance in predicting short-, medium-, and long-term survival outcomes of HF patients based on the evidence from the current study and data from previous similar studies (34). Therefore, we emphasize the future need to strengthen routine CRP monitoring in HF patients and simultaneously perform CALLY index assessments. To enhance precision in HF management, we recommend integrating the CALLY index into the clinical risk stratification system. Specific implementation includes: (1) establishing an automated assessment module within the information system to identify high-risk patients early based on index levels and optimize intervention strategies; and (2) combining it with existing risk models or artificial intelligence systems to enable dynamic and continuous prediction of mortality probability. Based on the results of the current study, we recommend classifying ADHF patients with a CALLY index of less than 3.14 as a high-risk population who require close monitoring of vital signs and appropriate interventions targeting nutrition and inflammation.

### Strengths and limitations

This study possesses several notable strengths: First, the research topic demonstrates innovation, as the CALLY index incorporates indicators that are readily accessible in clinical practice and derived from large-sample cohort data, endowing it with both clinical utility and translational potential for short-term prognosis assessment in ADHF patients. Second, a rigorous study design was implemented, encompassing a multidimensional validation strategy that includes subgroup analyses, temporal sensitivity testing, and data quality validation. This comprehensive approach substantially strengthens the robustness of our findings.

This study has several limitations: (1) While this study elucidated the prognostic utility of the baseline CALLY index for short-term mortality risk in ADHF patients, it did not explore the dynamic evolution of this index during hospitalization. Future research is recommended to focus on the temporal evolution characteristics of the CALLY index and evaluate its dynamic association with clinical prognosis. The specific approaches are as follows: (i) Increase the frequency of monitoring Alb, lymphocyte count, and CRP during hospitalization, and assess the trajectory of the CALLY index based on these repeated measurement data; (ii) Conduct regular follow-ups and perform trajectory analysis to assess the dynamic relationship between the CALLY index and clinical outcomes. (2) Although we adjusted for numerous potential confounders—including smoking/drinking status, comorbidities, and cardiac function—residual confounding may persist and potentially influence the results (87). (3) Since CRP is not routinely measured in clinical practice for HF patients, a relatively high proportion of participants were excluded due to missing CRP data, which may introduce a certain degree of selection bias. Although a comparison of baseline characteristics between those with and without missing data confirmed that the data were missing at random, the possibility of Missing Not at Random cannot be entirely ruled out. Large-scale multicenter cohort studies are therefore warranted to validate our results. (4) As a retrospective cohort analysis (non-interventional observational study design), this research inherently carries methodological limitations: First, the study framework cannot evaluate comparative clinical efficacy across various therapeutic regimens administered post-admission in ADHF patients. Second, observational data analysis only permits correlational inference between the CALLY index and outcomes, precluding the establishment of causal relationships between therapeutic interventions and clinical endpoints (88). These findings essentially reflect biomarker fluctuation patterns during the natural disease progression. (5) This study has geographical limitations: as the study population was predominantly recruited from Jiangxi Province in southern China, the generalizability of conclusions to geographically diverse regions in northern China and ethnically diverse populations requires confirmation through multicenter, cross-regional validation studies. Furthermore, as our study was conducted in a hospital-based setting, the generalizability of our findings may be limited to Chinese populations with similar healthcare-seeking behaviors. Differences in culture, diet, and healthcare systems between China and other regions may affect the applicability of our results to other populations. (6) A large number of subgroup analyses were conducted in the current study, which increases the risk of type I errors (i.e., false-positive findings). Although these analyses provide valuable insights for exploring potential effect modifiers, their results are based on a relatively small sample size and significant outcomes were only observed in the diabetes subgroup, and thus should be interpreted with caution and require further validation in independent cohorts.

## Conclusion

This study, based on the Jiangxi-ADHF II cohort, demonstrates that the CALLY index—a modified version of the PNI—serves as a robust prognostic tool for short-term outcomes in ADHF patients. A low CALLY index is significantly associated with an elevated risk of short-term mortality. Notably, the CALLY index outperforms the PNI in both mortality risk assessment and outcome prediction. Our findings underscore the potential clinical value of CALLY index evaluation for early risk stratification in ADHF populations, particularly among patients with comorbid diabetes.

## Data Availability

The raw data supporting the conclusions of this article will be made available by the authors, without undue reservation.

## References

[1] Lala A Hamo CE Bozkurt B Fiuzat M Blumer V Bukhoff D . Standardized definitions for evaluation of acute decompensated heart failure therapies: HF-ARC expert panel paper. JACC Heart Fail. (2024) 12:1–15. doi: 10.1016/j.jchf.2023.09.030, PMID: 38069997

[2] Njoroge JN Teerlink JR. Pathophysiology and therapeutic approaches to acute decompensated heart failure. Circ Res. (2021) 128:1468–86. doi: 10.1161/CIRCRESAHA.121.318186, PMID: 33983837 PMC8126502

[3] Habaybeh D de Moraes MB Slee A Avgerinou C. Nutritional interventions for heart failure patients who are malnourished or at risk of malnutrition or cachexia: a systematic review and meta-analysis. Heart Fail Rev. (2021) 26:1103–18. doi: 10.1007/s10741-020-09937-9, PMID: 32124164 PMC8310486

[4] Writing Committee Members; ACC/AHA Joint Committee Members. 2022 AHA/ACC/HFSA guideline for the management of heart failure. J Card Fail. (2022) 28:e1–e167. doi: 10.1016/j.cardfail.2022.02.010 35378257

[5] Hirose S Matsue Y Kamiya K Kagiyama N Hiki M Dotare T . Prevalence and prognostic implications of malnutrition as defined by GLIM criteria in elderly patients with heart failure. Clin Nutr. (2021) 40:4334–40. doi: 10.1016/j.clnu.2021.01.014, PMID: 33551220

[6] Li Y Zhu F Ren D Tong J Xu Q Zhong M . Establishment of in-hospital nutrition support program for middle-aged and elderly patients with acute decompendated heart failure. BMC Cardiovasc Disord. (2024) 24:259. doi: 10.1186/s12872-024-03887-y, PMID: 38762515 PMC11102219

[7] Driggin E Cohen LP Gallagher D Karmally W Maddox T Hummel SL . Nutrition assessment and dietary interventions in heart failure: JACC review topic of the week. J Am Coll Cardiol. (2022) 79:1623–35. doi: 10.1016/j.jacc.2022.02.025, PMID: 35450580 PMC9388228

[8] Agra Bermejo RM González Ferreiro R Varela Román A Gómez Otero I Kreidieh O Conde Sabarís P . Nutritional status is related to heart failure severity and hospital readmissions in acute heart failure. Int J Cardiol. (2017) 230:108–14. doi: 10.1016/j.ijcard.2016.12.067, PMID: 28038805

[9] McDonagh TA Metra M Adamo M Gardner RS Baumbach A Böhm M . 2021 ESC Guidelines for the diagnosis and treatment of acute and chronic heart failure: developed by the Task Force for the diagnosis and treatment of acute and chronic heart failure of the European Society of Cardiology (ESC). With the special contribution of the Heart Failure Association (HFA) of the ESC. Eur J Heart Fail. (2022) 24:4–131. doi: 10.1002/ejhf.2333 35083827

[10] Yoshihisa A Kanno Y Watanabe S Yokokawa T Abe S Miyata M . Impact of nutritional indices on mortality in patients with heart failure. Open Heart. (2018) 5:e000730. doi: 10.1136/openhrt-2017-000730, PMID: 29344381 PMC5761292

[11] Iida Y Kamiya K Adachi T Iwatsu K Kamisaka K Iritani N . Prognostic impact of nutrition measures in patients with heart failure varies with coexisting physical frailty. ESC Heart Fail. (2023) 10:3364–72. doi: 10.1002/ehf2.14519, PMID: 37675757 PMC10682846

[12] Buzby GP Mullen JL Matthews DC Hobbs CL Rosato EF. Prognostic nutritional index in gastrointestinal surgery. Am J Surg. (1980) 139:160–7. doi: 10.1016/0002-9610(80)90246-9, PMID: 7350839

[13] Liu CC Liu PH Chen HT Chen JY Lee CW Cheng WJ . Association of preoperative prognostic nutritional index with risk of postoperative acute kidney injury: a meta-analysis of observational studies. Nutrients. (2023) 15:2929. doi: 10.3390/nu15132929, PMID: 37447255 PMC10346508

[14] Zhang S Wang H Chen S Cai S Zhou S Wang C . Prognostic nutritional index and prognosis of patients with coronary artery disease: a systematic review and meta-analysis. Front Nutr. (2023) 10:1114053. doi: 10.3389/fnut.2023.1114053, PMID: 37006923 PMC10061069

[15] Zhang X Su Y. Low prognostic nutritional index predicts adverse outcomes in patients with heart failure: a systematic review and meta-analysis. Angiology. (2024) 75:305–13. doi: 10.1177/00033197231159680, PMID: 36826172

[16] Chen MY Wen JX Lu MT Jian XY Wan XL Xu ZW . Association between prognostic nutritional index and prognosis in patients with heart failure: a meta-analysis. Front Cardiovasc Med. (2022) 9:918566. doi: 10.3389/fcvm.2022.918566, PMID: 35757355 PMC9226429

[17] Zhang H Li D Li J. Prognostic significance of preoperative prognostic nutritional index in hepatocellular carcinoma after curative hepatectomy: a meta-analysis and systemic review. Front Nutr. (2024) 11:1433528. doi: 10.3389/fnut.2024.1433528, PMID: 39764415 PMC11700793

[18] Hung KC Chiu CC Hsu CW Ho CN Ko CC Chen IW . Association of preoperative prognostic nutritional index with risk of postoperative delirium: a systematic review and meta-analysis. Front Med. (2023) 9:1017000. doi: 10.3389/fmed.2022.1017000, PMID: 36698831 PMC9868631

[19] Núñez J Miñana G Bodí V Núñez E Sanchis J Husser O . Low lymphocyte count and cardiovascular diseases. Curr Med Chem. (2011) 18:3226–33. doi: 10.2174/092986711796391633 21671854

[20] Goonewardena SN Stein AB Tsuchida RE Rattan R Shah D Hummel SL. Monocyte subsets and inflammatory cytokines in acute decompensated heart failure. J Card Fail. (2016) 22:358–65. doi: 10.1016/j.cardfail.2015.12.014, PMID: 26705751 PMC4861694

[21] Chen D Assad-Kottner C Orrego C Torre-Amione G. Cytokines and acute heart failure. Crit Care Med. (2008) 36:S9–S16. doi: 10.1097/01.CCM.0000297160.48694.90, PMID: 18158483

[22] Garofalo M Corso R Tomasoni D Adamo M Lombardi CM Inciardi RM . Inflammation in acute heart failure. Front Cardiovasc Med. (2023) 10:1235178. doi: 10.3389/fcvm.2023.1235178, PMID: 38045909 PMC10690826

[23] Iida H Tani M Komeda K Nomi T Matsushima H Tanaka S . Superiority of CRP-albumin-lymphocyte index (CALLY index) as a non-invasive prognostic biomarker after hepatectomy for hepatocellular carcinoma. HPB. (2022) 24:101–15. doi: 10.1016/j.hpb.2021.06.414, PMID: 34244053

[24] Wu B Liu J Shao C Yu D Liao J. Integrating inflammation, nutrition, and immunity: the CALLY index as a prognostic tool in digestive system cancers—a systematic review and meta-analysis. BMC Cancer. (2025) 25:672. doi: 10.1186/s12885-025-14074-3, PMID: 40217204 PMC11992890

[25] Liu XY Zhang X Zhang Q Ruan GT Liu T Xie HL . The value of CRP-albumin-lymphocyte index (CALLY index) as a prognostic biomarker in patients with non-small cell lung cancer. Support Care Cancer. (2023) 31:533. doi: 10.1007/s00520-023-07997-9, PMID: 37610445

[26] Tsai YT Ko CA Chen HC Hsu CM Lai CH Lee YC . Prognostic value of CRP-albumin-lymphocyte (CALLY) index in patients undergoing surgery for oral cavity cancer. J Cancer. (2022) 13:3000–12. doi: 10.7150/jca.74930, PMID: 36046647 PMC9414026

[27] Lu Y Zhu X Xu Y Li Y Dai Q Chang X. Lower CALLY index levels indicate higher poor functional outcome risk in acute ischemic stroke patients treated with endovascular thrombectomy. Front Aging Neurosci. (2025) 17:1587861. doi: 10.3389/fnagi.2025.1587861, PMID: 40353064 PMC12061938

[28] Hu W Yuan Q Hu J Li M Xi Y Luo L. The association between C-reactive protein-albumin-lymphocyte index and depression in adults with type 2 diabetes mellitus: a cross-sectional study from NHANES. Psychoneuroendocrinology. (2025) 176:107442. doi: 10.1016/j.psyneuen.2025.107442, PMID: 40138851

[29] Zhang J Lin Y Zeng J Luo G Liao P Chen Q . The C-reactive protein (CRP)-albumin-lymphocyte (CALLY) index exhibits an L-shaped association with all-cause mortality in rheumatoid arthritis patients: a retrospective cohort study. BMC Rheumatol. (2025) 9:47. doi: 10.1186/s41927-025-00499-7, PMID: 40264172 PMC12013003

[30] Sarıdaş A Çetinkaya R. The prognostic value of the CALLY index in sepsis: a composite biomarker reflecting inflammation, nutrition, and immunity. Diagnostics. (2025) 15:1026. doi: 10.3390/diagnostics15081026, PMID: 40310418 PMC12025508

[31] Zhang J Zhao Q Liu S Yuan N Hu Z. Clinical predictive value of the CRP-albumin-lymphocyte index for prognosis of critically ill patients with sepsis in intensive care unit: a retrospective single-center observational study. Front Public Health. (2024) 12:1395134. doi: 10.3389/fpubh.2024.1395134, PMID: 38841671 PMC11150768

[32] Pan Y Wu TT Deng CJ Jiang ZH Yang Y Hou XG . Association between the C-reactive protein-albumin-lymphocyte (CALLY) index and adverse clinical outcomes in CAD patients after PCI: findings of a real-world study. Rev Cardiovasc Med. (2024) 25:111. doi: 10.31083/j.rcm2504111, PMID: 39076545 PMC11264017

[33] Ji H Luo Z Ye L He Y Hao M Yang Y . Prognostic significance of C-reactive protein-albumin-lymphocyte (CALLY) index after primary percutaneous coronary intervention in patients with ST-segment elevation myocardial infarction. Int Immunopharmacol. (2024) 141:112860. doi: 10.1016/j.intimp.2024.112860, PMID: 39142002

[34] He Q Cao Y Fan X Li B He Q Zhang H. Long-term prognostic value of CRP-albumin-lymphocyte index in elderly patients with heart failure with preserved ejection fraction. Exp Gerontol. (2025) 204:112744. doi: 10.1016/j.exger.2025.112744, PMID: 40179994

[35] Kursa M Rudnicki W. Feature selection with the Boruta package. J Stat Softw. (2010) 36:1–13. doi: 10.18637/jss.v036.i11

[36] Degenhardt F Seifert S Szymczak S. Evaluation of variable selection methods for random forests and omics data sets. Brief Bioinform. (2019) 20:492–503. doi: 10.1093/bib/bbx124, PMID: 29045534 PMC6433899

[37] Speiser JL Miller ME Tooze J Ip E. A comparison of random forest variable selection methods for classification prediction modeling. Expert Syst Appl. (2019) 134:93–101. doi: 10.1016/j.eswa.2019.05.028, PMID: 32968335 PMC7508310

[38] Adams KF Jr Fonarow GC Emerman CL LeJemtel TH Costanzo MR Abraham WT . Characteristics and outcomes of patients hospitalized for heart failure in the United States: rationale, design, and preliminary observations from the first 100,000 cases in the Acute Decompensated Heart Failure National Registry (ADHERE). Am Heart J. (2005) 149:209–16. doi: 10.1016/j.ahj.2004.08.005, PMID: 15846257

[39] Zhang YB Chen C Pan XF Guo J Li Y Franco OH . Associations of healthy lifestyle and socioeconomic status with mortality and incident cardiovascular disease: two prospective cohort studies. BMJ. (2021) 373:n604. doi: 10.1136/bmj.n604, PMID: 33853828 PMC8044922

[40] Rushton CA Satchithananda DK Jones PW Kadam UT. Non-cardiovascular comorbidity, severity and prognosis in non-selected heart failure populations: a systematic review and meta-analysis. Int J Cardiol. (2015) 196:98–106. doi: 10.1016/j.ijcard.2015.05.180, PMID: 26080284 PMC4518480

[41] Fried LP Ferrucci L Darer J Williamson JD Anderson G. Untangling the concepts of disability, frailty, and comorbidity: implications for improved targeting and care. J Gerontol A. (2004) 59:255–63. doi: 10.1093/gerona/59.3.m255, PMID: 15031310

[42] Sproston NR Ashworth JJ. Role of C-reactive protein at sites of inflammation and infection. Front Immunol. (2018) 9:754. doi: 10.3389/fimmu.2018.00754, PMID: 29706967 PMC5908901

[43] Matsumoto M Tsujino T Lee-Kawabata M Naito Y Sakoda T Ohyanagi M . Serum interleukin-6 and C-reactive protein are markedly elevated in acute decompensated heart failure patients with left ventricular systolic dysfunction. Cytokine. (2010) 49:264–8. doi: 10.1016/j.cyto.2009.11.006, PMID: 20005739

[44] Wang Z Li G Huang R Chang L Gong C Chen K . Prognostic value of fibrosis-5 index combined with C-reactive protein in patients with acute decompensated heart failure. BMC Cardiovasc Disord. (2023) 23:492. doi: 10.1186/s12872-023-03530-2, PMID: 37794360 PMC10552406

[45] Zhou J Du W Huang H Chen Y Chen L Zheng M. Association of CALLY index and CLR with COPD risk in middle-aged and older Americans: evidence from NHANES 2017–2020. Front Med. (2025) 12:1535415. doi: 10.3389/fmed.2025.1535415, PMID: 40313545 PMC12043464

[46] Liu Y Wei Y. Association between the CALLY index, vitamin D, and asthma: insights from NHANES. Front Allergy. (2025) 6:1557677. doi: 10.3389/falgy.2025.1557677, PMID: 40259948 PMC12009948

[47] Chen Y Liu M Zhang Y Yang X Yue M Chen X . Association between C-reactive protein-albumin-lymphocyte index and stroke: an NHANES analysis (1999–2010). Front Neurol. (2025) 16:1548666. doi: 10.3389/fneur.2025.1548666, PMID: 40242622 PMC12000103

[48] Huang D Wu H Huang Y. Novel indicator for erectile dysfunction: the CALLY index, evidence from data of NHANES 2001–2004. Front Endocrinol. (2025) 16:1527506. doi: 10.3389/fendo.2025.1527506, PMID: 40099259 PMC11911170

[49] Ye J Chen L Xu D Li R Lan R Chen S . Inverse association between CALLY index and angina pectoris in US adults: a population-based study. BMC Cardiovasc Disord. (2025) 25:94. doi: 10.1186/s12872-025-04544-8, PMID: 39934693 PMC11816972

[50] Wu L Han D Xue Y He S Ma Z Su S . Association between the C-reactive protein-albumin-lymphocyte index and metabolic syndrome: evidence from the 2003–2010 national health and nutrition examination survey. Diabetol Metab Syndr. (2025) 17:39. doi: 10.1186/s13098-025-01609-8, PMID: 39891279 PMC11783767

[51] Li Y Wei Q Ke X Xu Y Xu B Zhang K . Higher CALLY index levels indicate lower sarcopenia risk among middle-aged and elderly community residents as well as hospitalized patients. Sci Rep. (2024) 14:24591. doi: 10.1038/s41598-024-75164-z, PMID: 39426987 PMC11490578

[52] Xu Z Tang J Chen X Jin Y Zhang H Liang R. Associations of C-reactive protein-albumin-lymphocyte (CALLY) index with cardiorenal syndrome: insights from a population-based study. Heliyon. (2024) 10:e37197. doi: 10.1016/j.heliyon.2024.e37197 39296012 PMC11408039

[53] Güven B Deniz MF Geylan NA Kültürsay B Dönmez A Bulat Z . A novel indicator of all-cause mortality in acute coronary syndrome: the CALLY index. Biomark Med. (2025) 19:287–94. doi: 10.1080/17520363.2025.2483159, PMID: 40125936 PMC11980495

[54] Han D Wu L Zhou H Xue Y He S Ma Z . Associations of the C-reactive protein-albumin-lymphocyte index with all-cause and cardiovascular mortality among individuals with cardiovascular disease: evidence from the NHANES 2001–2010. BMC Cardiovasc Disord. (2025) 25:144. doi: 10.1186/s12872-025-04596-w, PMID: 40025412 PMC11874105

[55] Lang SQ Kong JJ Li GB Liu J. Prognostic value of CRP-albumin-lymphocyte index in patients with intrahepatic cholangiocarcinoma after radical resection. Front Med. (2025) 12:1543665. doi: 10.3389/fmed.2025.1543665, PMID: 40115790 PMC11922830

[56] Sakurai K Kubo N Hasegawa T Nishimura J Iseki Y Nishii T . Clinical significance of the CALLY index in patients with gastric cancer undergoing gastrectomy. World J Surg. (2024) 48:2749–59. doi: 10.1002/wjs.12357, PMID: 39349360

[57] Zhuang J Wang S Wang Y Wu Y Hu R. Prognostic value of CRP-albumin-lymphocyte (CALLY) index in patients undergoing surgery for breast cancer. Int J Gen Med. (2024) 17:997–1005. doi: 10.2147/IJGM.S447201, PMID: 38505146 PMC10949993

[58] Feng J Wang L Yang X Chen Q. Clinical significance of preoperative CALLY index for prognostication in patients with esophageal squamous cell carcinoma undergoing surgery. Sci Rep. (2024) 14:713. doi: 10.1038/s41598-023-51109-w, PMID: 38184747 PMC10771508

[59] Tsunematsu M Haruki K Taniai T Tanji Y Shirai Y Furukawa K . The impact of C-reactive protein-albumin-lymphocyte (CALLY) index on the prognosis of patients with distal cholangiocarcinoma following pancreaticoduodenectomy. Ann Gastroenterol Surg. (2022) 7:503–11. doi: 10.1002/ags3.12637, PMID: 37152771 PMC10154875

[60] Yang M Lin SQ Liu XY Tang M Hu CL Wang ZW . Association between C-reactive protein-albumin-lymphocyte (CALLY) index and overall survival in patients with colorectal cancer: from the investigation on nutrition status and clinical outcome of common cancers study. Front Immunol. (2023) 14:1131496. doi: 10.3389/fimmu.2023.1131496, PMID: 37063910 PMC10098202

[61] Ding Y Liu Y Yu J Cai C Fu L Zhu J . The association between the CALLY index and all-cause mortality in patients with COPD: results from the cohort study of NHANES 2007–2010. Int J Chron Obstruct Pulmon Dis. (2025) 20:159–69. doi: 10.2147/COPD.S485036, PMID: 39867991 PMC11766151

[62] Fernández-Pombo A Rodríguez-Carnero G Castro AI Cantón-Blanco A Seoane LM Casanueva FF . Relevance of nutritional assessment and treatment to counteract cardiac cachexia and sarcopenia in chronic heart failure. Clin Nutr. (2021) 40:5141–55. doi: 10.1016/j.clnu.2021.07.027, PMID: 34461588

[63] Yücel H Refiker Ege M Zorlu A Kaya H Beton O Güngör H . Lymphocytopenia is associated with poor NYHA functional class in chronic heart failure patients with reduced ejection fraction. Turk Kardiyol Dern Ars. (2015) 43:427–33. doi: 10.5543/tkda.2015.89439, PMID: 26148074

[64] Vaduganathan M Ambrosy AP Greene SJ Mentz RJ Subacius HP Maggioni AP . Predictive value of low relative lymphocyte count in patients hospitalized for heart failure with reduced ejection fraction: insights from the EVEREST trial. Circ Heart Fail. (2012) 5:750–8. doi: 10.1161/CIRCHEARTFAILURE.112.970525, PMID: 23051949

[65] Shantsila E Lip GY. Stroke in atrial fibrillation and improving the identification of ‘high-risk’ patients: the crossroads of immunity and thrombosis. J Thromb Haemost. (2015) 13:1968–70. doi: 10.1111/jth.13121, PMID: 26303061

[66] Arango Duque G Descoteaux A. Macrophage cytokines: involvement in immunity and infectious diseases. Front Immunol. (2014) 5:491. doi: 10.3389/fimmu.2014.00491, PMID: 25339958 PMC4188125

[67] Stumpf F Keller B Gressies C Schuetz P. Inflammation and nutrition: friend or foe? Nutrients. (2023) 15:1159. doi: 10.3390/nu15051159, PMID: 36904164 PMC10005147

[68] Eckart A Struja T Kutz A Baumgartner A Baumgartner T Zurfluh S . Relationship of nutritional status, inflammation, and serum albumin levels during acute illness: a prospective study. Am J Med. (2020) 133:713–722.e7. doi: 10.1016/j.amjmed.2019.10.031, PMID: 31751531

[69] Dent E Wright ORL Woo J Hoogendijk EO. Malnutrition in older adults. Lancet. (2023) 401:951–66. doi: 10.1016/S0140-6736(22)02612-5, PMID: 36716756

[70] Cohen S Danzaki K MacIver NJ. Nutritional effects on T-cell immunometabolism. Eur J Immunol. (2017) 47:225–35. doi: 10.1002/eji.201646423, PMID: 28054344 PMC5342627

[71] Ma YC Ju YM Cao MY Yang D Zhang KX Liang H . Exploring the relationship between malnutrition and the systemic immune-inflammation index in older inpatients: a study based on comprehensive geriatric assessment. BMC Geriatr. (2024) 24:19. doi: 10.1186/s12877-023-04604-8, PMID: 38178005 PMC10768166

[72] Charach G Grosskopf I Roth A Afek A Wexler D Sheps D . Usefulness of total lymphocyte count as predictor of outcome in patients with chronic heart failure. Am J Cardiol. (2011) 107:1353–6. doi: 10.1016/j.amjcard.2010.12.049, PMID: 21371686

[73] Nakamura K Miyoshi T Yoshida M Akagi S Saito Y Ejiri K . Pathophysiology and treatment of diabetic cardiomyopathy and heart failure in patients with diabetes mellitus. Int J Mol Sci. (2022) 23:3587. doi: 10.3390/ijms23073587, PMID: 35408946 PMC8999085

[74] Kenny HC Abel ED. Heart failure in type 2 diabetes mellitus. Circ Res. (2019) 124:121–41. doi: 10.1161/CIRCRESAHA.118.311371, PMID: 30605420 PMC6447311

[75] Frati G Schirone L Chimenti I Yee D Biondi-Zoccai G Volpe M . An overview of the inflammatory signalling mechanisms in the myocardium underlying the development of diabetic cardiomyopathy. Cardiovasc Res. (2017) 113:378–88. doi: 10.1093/cvr/cvx011, PMID: 28395009

[76] Vandanmagsar B Youm YH Ravussin A Galgani JE Stadler K Mynatt RL . The NLRP3 inflammasome instigates obesity-induced inflammation and insulin resistance. Nat Med. (2011) 17:179–88. doi: 10.1038/nm.2279, PMID: 21217695 PMC3076025

[77] Wen H Ting JP O'Neill LA. A role for the NLRP3 inflammasome in metabolic diseases—did Warburg miss inflammation? Nat Immunol. (2012) 13:352–7. doi: 10.1038/ni.2228, PMID: 22430788 PMC4090390

[78] Kawaguchi M Takahashi M Hata T Kashima Y Usui F Morimoto H . Inflammasome activation of cardiac fibroblasts is essential for myocardial ischemia/reperfusion injury. Circulation. (2011) 123:594–604. doi: 10.1161/CIRCULATIONAHA.110.982777, PMID: 21282498

[79] Dou L Jourde-Chiche N. Endothelial toxicity of high glucose and its by-products in diabetic kidney disease. Toxins. (2019) 11:578. doi: 10.3390/toxins11100578, PMID: 31590361 PMC6833015

[80] Pereira S Marliss EB Morais JA Chevalier S Gougeon R. Insulin resistance of protein metabolism in type 2 diabetes. Diabetes. (2008) 57:56–63. doi: 10.2337/db07-0887, PMID: 17940118

[81] Izzo A Massimino E Riccardi G Della Pepa G. A narrative review on sarcopenia in type 2 diabetes mellitus: prevalence and associated factors. Nutrients. (2021) 13:183. doi: 10.3390/nu13010183, PMID: 33435310 PMC7826709

[82] Bharucha AE Kudva YC Prichard DO. Diabetic gastroparesis. Endocr Rev. (2019) 40:1318–52. doi: 10.1210/er.2018-00161, PMID: 31081877 PMC6736218

[83] Zhang Y Wang T Kang DM. Nutritional status and influencing factors of elderly inpatients with diabetes. Chin J Gerontol. (2018) 38:2842–4.

[84] Tedeschi S Pilotti E Parenti E Vicini V Coghi P Montanari A . Serum adipokine zinc α2-glycoprotein and lipolysis in cachectic and noncachectic heart failure patients: relationship with neurohormonal and inflammatory biomarkers. Metabolism. (2012) 61:37–42. doi: 10.1016/j.metabol.2011.05.011, PMID: 21696792

[85] Zhang T Qin J Guo J Dong J Chen J Ma Y . Prevalence and influencing factors of malnutrition in diabetic patients: a systematic review and meta-analysis. J Diabetes. (2024) 16:e13610. doi: 10.1111/1753-0407.13610, PMID: 39364802 PMC11450603

[86] He W Yuan T Choezom D Hunkler H Annamalai K Lupse B . Ageing potentiates diet-induced glucose intolerance, β-cell failure and tissue inflammation through TLR4. Sci Rep. (2018) 8:2767. doi: 10.1038/s41598-018-20909-w, PMID: 29426925 PMC5807311

[87] Colditz GA. Overview of the epidemiology methods and applications: strengths and limitations of observational study designs. Crit Rev Food Sci Nutr. (2010) 50:10–2. doi: 10.1080/10408398.2010.526838, PMID: 21132580 PMC3024848

[88] Thiese MS. Observational and interventional study design types; an overview. Biochem Med. (2014) 24:199–210. doi: 10.11613/BM.2014.022, PMID: 24969913 PMC4083571

