# Supplementary material for: Evaluating the prognostic significance of the modified prognostic nutritional index—C-reactive protein-to-albumin-to-lymphocyte index in acute decompensated heart failure: special attention to the impact of diabetes
Source: Front Nutr. 2025 Nov 28;12:1636685. doi: 10.3389/fnut.2025.1636685 (PMC12698377; doi:10.3389/fnut.2025.1636685)
Supplement: Supplementary file 1 [file Table_1.DOCX]

Supplementary Table 1: Comparing baseline characteristics of participants with and without missing CRP.

|  | CRP | | *P*-value |
| --- | --- | --- | --- |
|  | Missing | Non-Missing |  |
| No. of subjects | 1551 | 1251 |  |
| Age (years) | 71.00 (62.00-79.00) | 71.00 (59.00-80.00) | 0.672 |
| LVEF (%) | 45.00 (35.00-55.00) | 49.00 (39.00-57.00) | <0.001 |
| WBC (×10^9^/L) | 6.10 (4.80-7.80) | 6.40 (5.06-8.51) | <0.001 |
| Neutrophil count(×10^9^/L) | 4.17 (3.10-5.75) | 4.53 (3.30-6.53) | <0.001 |
| Lymphocyte count(×10^9^/L) | 1.10 (0.79-1.51) | 1.00 (0.68-1.40) | <0.001 |
| Monocyte count(×10^9^/L) | 0.50 (0.39-0.64) | 0.50 (0.38-0.67) | 0.676 |
| RBC (×10^12^/L) | 4.09 (0.77) | 4.00 (0.80) | 0.074 |
| PLT (×10^9^/L) | 164.00 (126.00-210.00) | 164.00 (126.00-214.00) | 0.172 |
| Alb (g/L) | 35.92 (4.89) | 34.64 (5.22) | 0.091 |
| ALT (U/L) | 21.00 (14.00-36.00) | 22.00 (14.00-40.00) | 0.075 |
| AST (U/L) | 26.00 (19.00-38.00) | 27.00 (20.00-40.50) | 0.062 |
| Cr (umol/L) | 91.00 (71.00-126.00) | 88.00 (69.00-126.00) | 0.192 |
| BUN (mmol/L) | 7.48 (5.78-10.56) | 7.54 (5.62-10.83) | 0.922 |
| UA (umol/L) | 438.00 (349.00-546.00) | 419.00 (327.75-536.25) | 0.060 |
| FPG (mmol/L) | 5.30 (4.70-6.20) | 5.40 (4.70-6.40) | 0.232 |
| NT-proBNP (pmol/L) | 3743.00 (1997.00-6084.00) | 3531.00 (1713.50-6409.00) | 0.234 |
| Gender |  |  | 0.915 |
| Male | 897 (57.83%) | 726 (58.03%) |  |
| Female | 654 (42.17%) | 525 (41.97%) |  |
| Hypertension (n,%) |  |  | 0.356 |
| No | 857 (55.25%) | 713 (56.99%) |  |
| Yes | 694 (44.75%) | 538 (43.01%) |  |
| Diabetes (n,%) |  |  | 0.802 |
| No | 1157 (74.60%) | 928 (74.18%) |  |
| Yes | 394 (25.40%) | 323 (25.82%) |  |
| Stroke (n,%) |  |  | 0.131 |
| No | 1310 (84.46%) | 1030 (82.33%) |  |
| Yes | 241 (15.54%) | 221 (17.67%) |  |
| CHD (n,%) |  |  | 0.006 |
| No | 1023 (65.96%) | 886 (70.82%) |  |
| Yes | 528 (34.04%) | 365 (29.18%) |  |
| NYHA classification (n,%) |  |  | 0.227 |
| III | 1049 (67.63%) | 819 (65.47%) |  |
| IV | 502 (32.37%) | 432 (34.53%) |  |
| Drinking status |  |  | 0.689 |
| No | 1399 (90.20%) | 1134 (90.65%) |  |
| Yes | 152 (9.80%) | 117 (9.35%) |  |
| Smoking status |  |  | 0.996 |
| No | 1293 (83.37%) | 1043 (83.37%) |  |
| Yes | 258 (16.63%) | 208 (16.63%) |  |

Abbreviations: CHD: coronary heart disease; NYHA: New York Heart Association; LVEF: left ventricular ejection fraction; Cr: creatinine; WBC: white blood cell count; RBC: red blood cell count; PLT: platelet count; ALT: alanine aminotransferase; AST: aspartate aminotransferase; NT-proBNP: N-Terminal Pro-Brain Natriuretic Peptide; UA: uric acid; CRP: C reactive protein; Alb: albumin; FPG: fasting plasma glucose.

Supplementary Table 2: The missing number and rate of covariates.

|  | Non- Missing | Missing |
| --- | --- | --- |
| Gender | 1225 | 0 |
| Age | 1225 | 0 |
| Hypertension | 1225 | 0 |
| Diabetes | 1225 | 0 |
| Stroke | 1225 | 0 |
| CHD | 1225 | 0 |
| NYHA classification | 1225 | 0 |
| Drinking status | 1225 | 0 |
| Smoking status | 1225 | 0 |
| LVEF | 1179 | 46 |
| CRP | 1225 | 0 |
| WBC | 1225 | 0 |
| Neutrophil count | 1225 | 0 |
| Lymphocyte count | 1225 | 0 |
| Monocyte count | 1225 | 0 |
| PLT | 1225 | 0 |
| CALLY index | 1225 | 0 |
| PNI | 1225 | 0 |
| RBC | 1225 | 0 |
| Alb | 1225 | 0 |
| ALT | 1225 | 0 |
| AST | 1225 | 0 |
| Cr | 1214 | 11 |
| BUN | 1214 | 11 |
| UA | 1213 | 12 |
| FPG | 1181 | 44 |
| NT-proBNP | 1225 | 0 |

Abbreviations as in Table 1.

Supplementary Table 3: Collinearity screening between the CALLY index and covariates.

|  | VIF | | |
| --- | --- | --- | --- |
|  | Step 1 | Step 2 | Step 3 |
| CALLY index | 1.3 | 1.3 | 1.3 |
| Gender | 1.3 | 1.3 | 1.3 |
| Age | 1.4 | 1.4 | 1.4 |
| Hypertension | 1.2 | 1.2 | 1.2 |
| Diabetes | 1.4 | 1.4 | 1.4 |
| Stroke | 1.1 | 1.1 | 1.1 |
| CHD | 1.1 | 1.1 | 1.1 |
| NYHA classification | 1.2 | 1.2 | 1.2 |
| Drinking status | 1.5 | 1.5 | 1.4 |
| Smoking status | 1.5 | 1.5 | 1.5 |
| LVEF | 1.3 | 1.3 | 1.3 |
| CRP | 1.3 | 1.3 | 1.3 |
| WBC | 347.9 | NA | NA |
| Neutrophil count | 330.4 | 1.7 | 1.7 |
| Lymphocyte count | 13.3 | 1.5 | 1.5 |
| Monocyte count | 3.3 | 1.4 | 1.4 |
| PLT | 1.3 | 1.3 | 1.3 |
| RBC | 1.4 | 1.4 | 1.4 |
| Alb | 1.3 | 1.3 | 1.3 |
| ALT | 5.7 | 5.7 | NA |
| AST | 5.6 | 5.6 | 1.1 |
| Cr | 2.3 | 2.3 | 2.3 |
| BUN | 2.7 | 2.7 | 2.7 |
| UA | 1.7 | 1.6 | 1.6 |
| FPG | 1.5 | 1.4 | 1.4 |
| NT-proBNP | 1.3 | 1.3 | 1.3 |

VIF: variance inflation factor; VIF = 1/(1-R^2^). Abbreviations as in Table ​1.

Note: The variables with VIF>5 will be regarded as collinear variables.

Supplementary Table 4: Collinearity screening between the PNI and covariates.

|  | VIF | | | |
| --- | --- | --- | --- | --- |
|  | Step 1 | Step 2 | Step 3 | Step 4 |
| PNI | 557327.9 | 2.1 | 2.1 | 2.1 |
| Gender | 1.3 | 1.3 | 1.3 | 1.3 |
| Age | 1.4 | 1.4 | 1.4 | 1.4 |
| Hypertension | 1.2 | 1.2 | 1.2 | 1.2 |
| Diabetes | 1.4 | 1.4 | 1.4 | 1.4 |
| Stroke | 1.1 | 1.1 | 1.1 | 1.1 |
| CHD | 1.1 | 1.1 | 1.1 | 1.1 |
| NYHA classification | 1.2 | 1.1 | 1.1 | 1.1 |
| Drinking status | 1.5 | 1.5 | 1.5 | 1.4 |
| Smoking status | 1.5 | 1.5 | 1.5 | 1.5 |
| LVEF | 1.3 | 1.3 | 1.3 | 1.3 |
| CRP | 1.3 | 1.3 | 1.3 | 1.2 |
| WBC | 347.8 | 347.6 | NA | NA |
| Neutrophil count | 330.3 | 330.1 | 1.7 | 1.7 |
| Lymphocyte count | 117054.8 | 13.7 | 2 | 2 |
| Monocyte count | 3.3 | 3.3 | 1.4 | 1.4 |
| PLT | 1.3 | 1.3 | 1.3 | 1.3 |
| RBC | 1.4 | 1.4 | 1.4 | 1.4 |
| Alb | 349264.5 | NA | NA | NA |
| ALT | 5.7 | 5.7 | 5.7 | NA |
| AST | 5.6 | 5.6 | 5.6 | 1.1 |
| Cr | 2.3 | 2.3 | 2.3 | 2.3 |
| BUN | 2.7 | 2.7 | 2.7 | 2.7 |
| UA | 1.6 | 1.6 | 1.6 | 1.6 |
| FPG | 1.5 | 1.5 | 1.4 | 1.4 |
| NT-proBNP | 1.3 | 1.3 | 1.3 | 1.3 |

VIF: variance inflation factor; VIF = 1/(1-R^2^). Abbreviations as in Table ​1.

Note: The variables with VIF>5 will be regarded as collinear variables.

Supplementary Table 5: Predictive performance of the CALLY index versus PNI model.

|  | PNI model | CALLY Index Model | improve | *P*-value |
| --- | --- | --- | --- | --- |
| AUC | 0.74 | 0.80 | 0.06 | 0.03 |
| continuous-NRI | - | - | 0.21 (0.11, 0.35) | <0.01 |

AUC: area under the curve; NRI: net reclassification improvement; other abbreviations as in Table ​1.

Supplementary Table 6: Predictive Performance of the CALLY Index versus ADHERE model.

|  | ADHERE model | CALLY Index Model | improve | *P*-value |
| --- | --- | --- | --- | --- |
| AUC | 0.61 | 0.80 | 0.19 | <0.01 |
| continuous-NRI | - | - | 0.29 (0.18, 0.41) | <0.01 |

AUC: area under the curve; NRI: net reclassification improvement; other abbreviations as in Table ​1.

Supplementary Table 7: ROC analysis assessed the CALLY index's predictive value for 30-day mortality in ADHF patients >65 years with either preserved or reduced ejection fraction.

|  | AUC | 95%CI low | 95%CI upp | Best threshold | Specificity | Sensitivity |
| --- | --- | --- | --- | --- | --- | --- |
| LVEF<50 |  |  |  |  |  |  |
| CALLY index | 0.73 | 0.64 | 0.81 | 1.67 | 0.66 | 0.73 |
| LVEF≥50 |  |  |  |  |  |  |
| CALLY index | 0.82 | 0.74 | 0.89 | 1.17 | 0.71 | 0.80 |

Abbreviations: AUC: area under the curve; CALLY: C-reactive protein-to-albumin-to-lymphocyte; ROC: receiver operating characteristic curve; ADHF: acute decompensated heart failure.
